# Interaction of Hypertension and Diabetes Mellitus on Post-Cardiac Arrest Treatments and Outcomes in Cancer Patients Following Out-of-Hospital Cardiac Arrest

**DOI:** 10.3390/jcm14145088

**Published:** 2025-07-17

**Authors:** Jungho Lee, Dahae Lee, Eujene Jung, Jeong Ho Park, Young Sun Ro, Sang Do Shin, Hyun Ho Ryu

**Affiliations:** 1 Department of Emergency Medicine, Chonnam National University Hospital, 42 Jebong-ro, Dong-gu, Gwangju 61469, Republic of Korea; sw97220501@naver.com (J.L.); dhlee115@naver.com (D.L.); 2 Department of Emergency Medicine, Chonnam National University Medical School, 42 Jebong-ro, Dong-gu, Gwangju 61469, Republic of Korea; 3 Department of Emergency Medicine, Seoul National University College of Medicine, 101 Daehak-ro, Jongno-gu, Seoul 03080, Republic of Korea; timthe@gmail.com (J.H.P.);; 4 Laboratory of Emergency Medical Services, Seoul National University Hospital Biomedical Research Institute, 101 Daehak-ro, Jongno-gu, Seoul 03080, Republic of Korea; em.ro.youngsun@gmail.com

**Keywords:** cancer, out-of-hospital cardiac arrest, hypertension, diabetes mellitus, percutaneous coronary intervention, targeted temperature management, post-resuscitation treatment

## Abstract

**Background/Objectives**: Out-of-hospital cardiac arrest (OHCA) is associated with high mortality, and outcomes may be influenced by underlying conditions such as cancer, hypertension (HTN), and diabetes mellitus (DM). This study aimed to evaluate whether HTN and DM modify the effects of post-resuscitation treatments—specifically targeted temperature management (TTM) and percutaneous coronary intervention (PCI)—on survival and neurological recovery in OHCA patients with a history of cancer. **Methods**: This retrospective cohort study analyzed data from the Korean national OHCA registry between January 2018 and December 2021. Adults aged ≥18 years with presumed cardiac-origin OHCA and a documented history of cancer—defined as any prior cancer diagnosis recorded in medical records regardless of remission status—were included. Multivariable logistic regression was used to examine associations between treatment and outcomes, and interaction effects were assessed using adjusted *p*-values to account for multiple testing. **Results**: Among the 124,916 EMS-assessed OHCA cases, 4115 patients met the inclusion criteria. TTM and PCI were both statistically associated with good neurological recovery (TTM: adjusted odds ratio [aOR], 1.69; 95% confidence interval [CI], 1.12–2.55; *p* < 0.05; PCI: aOR, 11.35; 95% CI, 7.98–16.14; *p* < 0.05). In interaction analyses, the benefit of TTM and PCI for achieving good neurological recovery was attenuated in patients with diabetes mellitus (DM; TTM: aOR, 0.59; 95% CI, 0.23–1.49; PCI: aOR, 4.94; 95% CI, 2.69–9.06) and hypertension (HTN; TTM: aOR, 0.94; 95% CI, 0.49–1.82; PCI: aOR, 7.47; 95% CI, 4.48–12.44), with adjusted *p*-values < 0.05 for all interactions. **Conclusions**: In OHCA patients with a history of cancer, TTM and PCI are associated with improved survival and neurological outcomes. However, the presence of comorbidities such as HTN and DM may attenuate these benefits. These findings support the need for individualized post-resuscitation care strategies that account for comorbid conditions.

## 1. Introduction

Out-of-hospital cardiac arrest (OHCA) is recognized as a societal burden due to the associated rates of mortality and disability [1]. Although survival rates have improved over the past few decades, the global incidence of emergency medical service (EMS)-treated OHCAs is approximately 30–90 per 100,000 population, with an overall mortality rate of 84–95% [2,3]. Much research has focused on identifying factors that improve survival after cardiac arrest. Prehospital factors such as age, bystander status, bystander cardiopulmonary resuscitation (CPR), early defibrillation, and ambulance response time have been reported to be associated with survival [4,5,6]. In hospital factors such as immediate coronary angiography (CAG) with percutaneous coronary intervention (PCI) and post-resuscitation treatment strategies including targeted temperature management (TTM) have also been shown to affect survival [7,8]. Evidence of post-resuscitation treatment strategies that improve OHCA survival includes immediate coronary angiography (CAG) with percutaneous coronary intervention (PCI) and targeted temperature management (TTM), which have been reported in multiple studies [8,9].

As the global population expands and life expectancies increase, the number of cancer patients and the global burden of cancer also grow [10]. Given that advances in cancer detection and treatment are improving survival and that the risk of OHCA increases with age, the likelihood that a patient experiencing an OHCA will have a history of cancer also increases [11,12]. However, several studies have reported that patients with a history of cancer who experience a return of spontaneous circulation (ROSC) are less likely to receive post-resuscitation treatments, such as PCI or TTM, compared to patients with no cancer history [13,14,15]. This discrepancy, possibly driven by concerns about poor prognosis or treatment futility even in inactive malignancies, underscores the importance of identifying cancer patient subgroups who may benefit from aggressive post-arrest interventions, particularly given that comorbidities often lead to the interruption of such treatments in cancer patients more than in non-cancer patients [12].

Comorbidities such as hypertension (HTN) and diabetes mellitus (DM) are frequently present in cancer patients and are known to increase the risk of cardiovascular disease and cardiac arrest [16,17,18,19]. More recently, attention has shifted to how these comorbidities may modify the effectiveness of post-resuscitation interventions. For instance, HTN has paradoxically been associated with improved survival following OHCA, possibly due to higher baseline perfusion pressures or medication-related protective effects [20]. Conversely, DM has been linked to attenuated neuroprotective effects of TTM, likely due to impaired glycemic control, oxidative stress, and vascular dysfunction [21,22,23]. These complex and sometimes contradictory interactions underscore the need for a more nuanced understanding of patient-specific responses to treatment.

Therefore, this study aimed to evaluate the association between HTN and DM and the outcomes of post-resuscitation treatments among cancer patients who experienced OHCA. Using a nationwide, population-based OHCA registry in South Korea, the analysis focused on whether these comorbidities modified the effectiveness of TTM and PCI, with the ultimate goal of informing more individualized, evidence-based post-cardiac arrest care for cancer survivors.

## 2. Materials and Methods

### 2.1. Study Design and Data Source

This cross-sectional observational study utilized data from the national OHCA registry from January 2018 to December 2021. The registry was developed in 2006 by the Fire Service and the Centers for Disease Control and Prevention and contains data on OHCA patients treated by the Korean EMS from ambulance dispatch sheets, Utstein factors, and the In-Depth Cardiac Arrest Ambulance Registry. Hospital treatment data, including use of post-resuscitation and survival, are collected via retrospective review of medical records by trained reviewers. Quality control is performed monthly, and feedback is provided to medical record reviewers to ensure data quality [24].

This study was approved by the Institutional Review Board of Seoul National University Hospital (IRB No. SNUH-1103-153-357), which waived the requirement for informed consent.

### 2.2. Study Setting

The Korean EMS system is operated by the national fire department and has 18 provincial headquarters, 219 stations, 1055 safety centers, and 1420 ambulances. All ambulances offer Basic Life Support (BLS) to OHCA patients at the scene by certified EMS providers, including level 1 and level 2 emergency medical technicians and registered nurses, following the EMS CPR protocol. Only a doctor can declare death in the Korean system; hence, all OHCA patients are transported to the nearest hospital emergency department (ED).

EDs in South Korea are categorized into three levels (1 to 3) by the government, based on factors such as human resources, essential equipment, level of service, and size of department. Each classification is re-evaluated annually. There are 40 level 1 emergency department (EDs), approximately 130 level 2 EDs, and 240 level 3 EDs. Emergency physicians provide advanced emergency care services in level 1 or 2 EDs 24 h a day. Level 3 EDs are staffed by general physicians and provide basic emergency care.

### 2.3. Study Population

Patients were selected from the national OHCA registry between January 2018 and December 2021. The study population included adults aged 18 years or older who experienced an OHCA of presumed cardiac origin and had a documented history of cancer. The history of cancer was defined as any cancer diagnosis recorded in the patient’s medical records, as identified by professional reviewers from the Korea Centers for Disease Control and Prevention during systematic chart abstraction. The study did not distinguish between active cancer and remission status.

**Figure 1:**
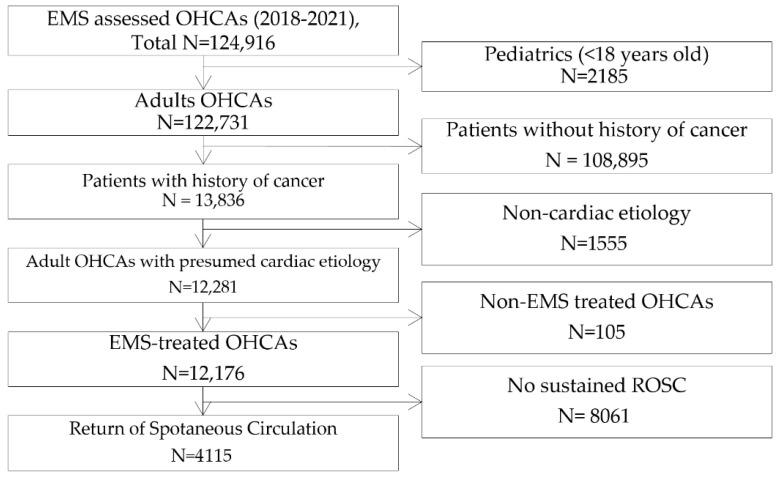
Patient flow chart. OHCA—out-of-hospital cardiac arrest, ROSC—return of spontaneous circulation.

Patients were included if they achieved sustained ROSC and had available records indicating the presence or absence of post-resuscitation treatments such as PCI and TTM. Patients were excluded if they were younger than 18 years, had a noncardiac cause of arrest such as trauma, drowning, or poisoning, were not treated by EMS, or did not achieve sustained ROSC. Post-resuscitation treatments could not be administered in patients who did not achieve sustained ROSC and were therefore excluded. Among 124,916 OHCA cases identified during the study period, a total of 4115 patients met the eligibility criteria and were included in the final analysis (Figure 1).

### 2.4. Outcome Measures

This study’s primary outcome is good neurological recovery in post-resuscitation patients. Good neurological recovery is defined as achieving a cerebral performance category (CPC) score of 1 (indicating good recovery) or 2 (signifying mild disability) at the time of hospital discharge [25]. The secondary outcome is survival to hospital discharge. These data are analyzed in the context of patient comorbidities and the post-resuscitation treatment received. Outcome data were collected by trained medical record reviewers from medical records documented by physicians.

### 2.5. Measurements

Professional medical record reviewers, employed by the Korean Centers for Disease Control and Prevention (CDC), visited multiple hospitals to digitally gather patient records. These records include information on whether a patient has a history of cancer and demographic data (patient age; gender; history of DM, HTN, cardiac comorbidities, stroke, or kidney disease; area of residence). We also noted the following characteristics of the incident: whether the cardiac arrest happened in a public or private setting, whether the event was witnessed, whether bystander CPR was performed, the shockable heart rhythm detected by EMS, response time interval (from the emergency call to EMS arrival), scene time interval (time spent at the scene), transport time interval (the duration of EMS transport from the scene to the emergency department), whether a multi-tier response system was implemented, the type of airway management used by EMS (advanced airway, bag-valve mask, or none), ED level (1 or 2), post-resuscitation treatment performed (PCI or TTM), and patient clinical outcomes.

### 2.6. Statistical Analysis

Categorical variables were reported as numbers and percentages (%) and analyzed by Chi-square tests. Multivariable logistic regression analysis was performed to estimate the adjusted odds ratios (aORs) and 95% confidence intervals (CIs) for clinical outcomes of post-resuscitation treatments in OHCA patients with a history of cancer. We considered all possible confounding factors, including demographics (age, gender, comorbidities), community factors (residential area, occurrence in a private place), and shockable rhythm at EMS.

To assess whether the effects of post-resuscitation treatments varied by comorbidities, interaction terms between comorbidities (hypertension and diabetes mellitus) and treatments (PCI and TTM) were included in the regression models. Given the multiple comparisons inherent in these interaction analyses, we applied the Benjamini–Hochberg procedure to control the false discovery rate (FDR) and reduce the likelihood of type I errors [26]. A *p*-value < 0.05 was considered to be statistically significant. All data were analyzed by using SAS 9.4 (SAS Institute Inc., Cary, NC, USA).

## 3. Results

### 3.1. Demographic Characteristics

Among 124,916 EMS-assessed OHCAs in South Korea during the study period, 4115 patients were included in the analysis. Excluded were patients younger than 18 years of age (*n* = 2185), patients without a history of cancer (*n* = 108,895), noncardiac etiology (*n* = 1555), non-EMS-treated OHCAs (*n* = 105), and no sustained ROSC (*n* = 8061) (Figure 1).

Among the 4115 included patients, 302 (7.3%) received TTM. Compared with patients not receiving TTM, the TTM group had a younger age profile (41.4% aged 19–65, *p* < 0.01) and higher incidences of HTN (46.7%, *p* < 0.01), cardiac comorbidities (26.2%, *p* < 0.01), and shockable rhythms at EMS (29.8%, *p* < 0.01). They were frequently from metropolitan areas (58.9%, *p* < 0.01), received multi-tier responses, and higher-level ED care (99.4% vs. 81.3%, *p* < 0.01). Patients who underwent TTM were more likely to receive additional treatments, such as ECMO (2.6% vs. 1.0%, *p* < 0.01) or PCI (25.8% vs. 6.4%, *p* < 0.01), and were associated with more positive outcomes, including survival to hospital discharge (38.1% vs. 10.6%, *p* < 0.01) and good neurological recovery (16.6% vs. 5.4%, *p* < 0.01) (Table 1).

Among the 4115 included patients, 323 (7.8%) underwent PCI. Compared with patient not receiving PCI, the PCI group had a younger age profile (46.1% aged 19–65, *p* < 0.01) and higher incidences of DM (36.8% vs. 27.4%, *p* < 0.01), HTN (47.4% vs. 36.8%, *p* < 0.01), and cardiac comorbidities (32.2% vs. 16.9%, *p* < 0.01). They frequently experienced a cardiac arrest in public (52.3%, *p* < 0.01), received bystander CPR (62.8%, *p* < 0.01), and presented with shockable rhythms (57.6%, *p* < 0.01). Patients who underwent TTM were more likely to receive additional treatments, such as ECMO (11.1% vs. 0.2%, *p* < 0.01) or TTM (24.1% vs. 5.9%, *p* < 0.01). The PCI group experienced significantly higher rates of survival to hospital discharge (59.1% vs. 8.7%, *p* < 0.01) and good neurological recovery (45.2% vs. 2.8%, *p* < 0.01) (Table 2).

### 3.2. Main Analysis

#### 3.2.1. Effects of TTM and PCI

Compared to non-TTM and non-PCI groups, the TTM and PCI groups were associated with significantly higher aORs for survival to hospital discharge and good neurological recovery. For survival to discharge, the TTM and PCI groups had aORs of 3.91 (95% CI: 2.92–5.22, *p*-value < 0.05) and 7.95 (95% CI: 5.97–10.57, *p*-value < 0.05), respectively. For good neurological outcome, they had aORs of 1.69 (95% CI: 1.12–2.55, *p*-value < 0.05) and 11.35 (95% CI: 7.98–16.14, *p*-value < 0.05) (Table 3).

#### 3.2.2. Effects of DM and HTN

Patients with diabetes showed slightly lower rates of survival to hospital discharge (12.44%) compared to those without DM (12.75%). Similarly, the rate of good neurological recovery was slightly lower in patients with DM (5.70%) than in those without DM (6.36%). However, these differences were not statistically significant (survival to discharge: aOR 0.93, 95% CI: 0.73–1.19; good neurological recovery: aOR 1.00, 95% CI: 0.69–1.43).

In contrast, patients with HTN demonstrated better outcomes compared to those without HTN. The survival to hospital discharge rate was higher in patients with HTN (14.60%) compared to those without HTN (11.49%), and this difference was statistically significant (aOR 1.46, 95% CI: 1.16–1.83, *p*-value < 0.05). Similarly, patients with HTN had a higher rate of good neurological recovery (6.65%) compared to those without HTN (5.88%), with a statistically significant adjusted odds ratio (aOR 1.48, 95% CI: 1.06–2.06, *p*-value < 0.05) (Table 4).

### 3.3. Interaction Analysis

Among patients who received TTM, those without DM had an aOR of 4.91 (95% CI: 3.49–6.91) for survival to hospital discharge, while those with DM had an aOR of 2.20 (95% CI: 1.26–3.86). Similarly, patients without HTN had an aOR of 6.05 (95% CI: 4.10–8.91), and those with HTN had an aOR of 2.31 (95% CI: 1.48–3.58). The interaction between comorbidity status and the effect of TTM on survival was statistically significant for both DM and HTN (FDR-adjusted *p* < 0.05).

For good neurological recovery, non-DM patients in the TTM group had an aOR of 2.32 (95% CI: 1.46–3.69), compared to 0.59 (95% CI: 0.23–1.49) in patients with DM. Patients with HTN had an aOR of 2.58 (95% CI: 1.52–4.38), while those without HTN had an aOR of 0.94 (95% CI: 0.49–1.82). Both interactions were statistically significant (FDR-adjusted *p* < 0.05).

Among PCI group patients, those without DM had an aOR of 10.33 (95% CI: 7.23–14.76) for survival to hospital discharge, while those with DM had an aOR of 5.00 (95% CI: 3.14–7.96), indicating a statistically significant interaction (FDR-adjusted *p* < 0.05). For HTN, patients without the condition had an aOR of 9.97 (95% CI: 6.77–14.68) and those with HTN had an aOR of 6.18 (95% CI: 4.13–9.25); however, the interaction was not statistically significant (FDR-adjusted *p* = 0.16).

Regarding neurological outcomes, non-DM patients in the PCI group had an aOR of 16.45 (95% CI: 10.80–25.05), compared to 4.94 (95% CI: 2.69–9.06) in DM patients. Non-HTN patients had an aOR of 15.77 (95% CI: 9.94–25.02), while HTN patients had an aOR of 7.47 (95% CI: 4.48–12.44). Both interactions were statistically significant (FDR-adjusted *p* < 0.05) (Table 5).

## 4. Discussion

This study found that post-resuscitation treatments (TTM and PCI) are effective post-resuscitation treatments for patients with a history of cancer, comorbidities present before the cardiac arrest (HTN or DM), which affect the efficacy of these treatments on neurological recovery in cancer survivors of OHCA with a presumed cardiac cause. TTM was not found to confer a significant benefit on good neurological recovery in patients with HTN or DM. These results suggest that the history of these comorbidities alters the efficacy of TTM on neurological outcomes in OHCA patients. The effectiveness of PCI on neurological recovery was also attenuated in patients with HTN or DM. These results showed that in the population of cancer survivors who experience OHCA, DM, and HTN are not poor prognostic factors, but they each modified the effects of TTM and PCI.

Previous studies of post-resuscitation treatments for cancer survivors have primarily focused on comparing the proportion of patients receiving treatments and outcomes with those of non-cancer patients. In a Korean study, TTM was reported to be less effective in cancer patients than non-cancer patients for both survival to hospital discharge and good neurological recovery, whereas PCI showed no statistical significance [13]. By limiting our study to cancer patients, we were able to objectively verify the effectiveness of each treatment, and the data show that each was effective.

In contrast to the overall negative impact of diabetes mellitus (DM) and hypertension (HTN) on the mortality of cancer survivors [16,17], this study reveals a different trend in the context of post-cardiac arrest outcomes in cancer survivors. Although DM does not significantly influence survival or neurological recovery, HTN is associated with better outcomes after cardiac arrest. This finding aligns with observations in the general population, where HTN has similarly been linked to improved post-resuscitation prognosis [20]. These reports suggest that HTN might confer protective effects in post-resuscitation scenarios. Some studies suggest that antihypertensive medication may play an important role in preserving ischemic preconditioning, preventing cardiac remodeling, and reducing the recurrence of myocardial infarction and ischemia [27], but the benefit of these effects, particularly in cancer patients on chemotherapy, is controversial and warrants further study [28,29].

Previous studies observed that DM has a negative impact on neurological recovery in patients receiving TTM [21], a conclusion that is supported by this study. Optimal glycemic control during TTM is considered crucial for neurological recovery [22]. However, a history of DM complicates this control due to increased oxidative stress and protein glycosylation, decreased blood insulin levels following cardiac arrest, and the risk of hypoglycemia during the rewarming phase [22,23]. These factors are particularly challenging in diabetic patients and diminish the efficacy of TTM. The impact of DM on the outcomes in patients receiving PCI is controversial, although some studies suggest a poorer prognosis [30,31]. The effect of DM on PCI efficacy may be due to increased platelet reactivity and vulnerability of the coronary arteries in DM patients, leading to a lower coronary reserve [32].

The role of HTN in the post-resuscitation phase of cancer survivors experiencing an OHCA is complex and multifaceted. This is because HTN has been shown to have a positive impact on OHCA patient prognosis, but also modifies the efficacy of treatments such as PCI and TTM, particularly with regard to neurological recovery. A previous study of the interaction between HTN and TTM in the general population also found this trend [33]. Persistent high blood pressure can lead to changes in the cerebral artery structure and function, resulting in a reduction in cerebral blood flow (CBF) [34]. Because maintaining CBF after ROSC is crucial for survival, blood pressure regulation during TTM is emphasized [35]. However, cerebral blood vessels stiffen in patients with longstanding HTN, and this stiffness can interfere with the goals of TTM, which include increasing CBF and protecting the blood–brain barrier. Moreover, HTN can negatively impact cardiac function, leading to issues such as heart failure and left ventricular hypertrophy, which in turn may lower cardiac output after cardiac arrest [36,37]. A reduction in cardiac output makes it challenging to maintain sufficient cerebral perfusion pressure during post-resuscitation treatments, possibly leading to poorer neurological outcomes. In addition, this trend is more pronounced in patients requiring post-resuscitation treatments, suggesting that it may be exacerbated in more critical patients, but further research is needed.

Comorbidities such as DM and HTN in cancer patients are known to exacerbate these pathophysiologic conditions and increase the risk of CVD, the most common cause of OHCA [38,39]. Cancer elevates cardiovascular risks through systemic inflammation, immunosuppression agents, and therapy-induced cardiac toxicity [40,41]. DM and HTN are prevalent in cancer survivors and further compound these risks by accelerating coronary artery disease, causing electrophysiological disturbances, and contributing to myocardial remodeling [19]. This synergy of cancer pathophysiology with DM and HTN not only increases cardiac event risks but may also impact the efficacy of post-resuscitation treatments like TTM and PCI. Recent evidence also suggests that gut microbiota may contribute to the development of hypertension and diabetes through chronic inflammation and metabolic dysregulation, providing a novel perspective on the pathophysiology of these comorbidities [42]. Understanding these underlying mechanisms may help explain variability in treatment response and support the development of more tailored post-resuscitation strategies in this population.

This study has several limitations. First, it is a population-based observational study, not an interventional one, potentially containing significant unconsidered biases. Second, the collected data contained no information on the duration, severity, or treatment modalities for patients with HTN and DM. The collected data also did not include current cancer treatments and therapies received, which might have influenced the study outcomes. Third, blood pressure data during TTM and PCI were not collected, making it difficult to ascertain the impact of pre-cardiac arrest diagnoses of HTN and DM. Fourth, the prevalence of HTN and DM may have been underestimated based on medical record reviews in the study population that survived to hospital admission. Fifth, outcomes of post-cardiac arrest care are also related to pre-cardiac arrest CPC scores, but pre-cardiac arrest CPC scores were not measured in this study. Cancer patients, who often have multiple comorbidities and are likely to have lower CPC scores, may have received less intensive post-cardiac arrest treatments such as TTM and PCI. This unmeasured factor should be considered a limitation of the study. Sixth, our dataset did not include information on prehospital pharmacologic interventions such as epinephrine administration, which have been shown to influence ROSC and post-resuscitation outcomes in previous studies [43]. Although our study focused on hospital-based treatments following resuscitation, future research should aim to integrate both prehospital and in-hospital management strategies to better understand their combined impact on outcomes in OHCA patients with cancer. Finally, changes in the TTM and PCI protocols, such as method, target temperature, duration of cooling, and PCI techniques during the study period, were not accounted for in the analysis.

## 5. Conclusions

For cancer survivors who experience OHCA, post-resuscitation treatments such as TTM and PCI are associated with improved survival and neurological outcomes. However, the effectiveness of these treatments was attenuated in patients with pre-existing HTN or DM, especially regarding good neurological recovery. These findings highlight the importance of individualized treatment planning that accounts for comorbid conditions in post-arrest care. Further research is warranted to validate these findings and guide clinical decision-making.

## Figures and Tables

**Table 1:** Baseline characteristics by target temperature management (TTM).

		Total	TTM		
			Yes	No	*p*-Value
		N (%)		N (%)	
Total		4115 (100.0)	302 (100.0)	3813 (100.0)	
Age					<0.01
	19–65	1174 (28.5)	125 (41.4)	1049 (27.5)	
	65–	2941 (71.5)	177 (58.6)	2764 (72.5)	
Gender					0.92
	Female	1333 (32.4)	97 (32.1)	1236 (32.4)	
Comorbidity					
	Diabetes	1158 (28.1)	91 (30.1)	1067 (28.0)	0.42
	Hypertension	1548 (37.6)	141 (46.7)	1407 (36.9)	<0.01
	Cardiac comorbidities	745 (18.1)	79 (26.2)	666 (17.5)	<0.01
	Stroke	309 (7.5)	24 (7.9)	285 (7.5)	0.76
	Kidney disease	311 (7.6)	39 (12.9)	272 (7.1)	0.10
Metropolis					<0.01
	Yes	1876 (45.6)	178 (58.9)	1698 (44.5)	
Place of arrest					0.60
	Public	1509 (36.7)	115 (38.1)	1394 (36.6)	
Arrest witnessed					0.95
	Yes	2759 (67.0)	202 (66.9)	2557 (67.1)	
Bystander CPR					0.19
	Yes	2043 (49.6)	161 (53.3)	1882 (49.4)	
Shockable rhythm at EMS					<0.01
	Shockable	536 (13.0)	90 (29.8)	446 (11.7)	
Response time interval					0.03
	~5 min	926 (22.5)	71 (23.5)	855 (22.4)	
	6~8 min	1627 (39.5)	137 (45.4)	1490 (39.1)	
	9 min~	1562 (38.0)	94 (31.1)	1468 (38.5)	
	Mean (SD)	8.5 (4.4)	7.7 (3.1)	8.5 (4.5)	
Scene time interval					0.91
	~10 min	1097 (26.7)	80 (26.5)	1017 (26.7)	
	11~15 min	1429 (34.7)	102 (33.8)	1327 (34.8)	
	16 min~	1589 (38.6)	120 (39.7)	1469 (38.5)	
	Mean (SD)	14.5 (6.8)	14.7 (6.5)	14.5 (6.8)	
Transport time interval					0.05
	~4 min	1524 (37.0)	98 (32.5)	1426 (37.4)	
	5~7 min	1518 (36.9)	131 (43.4)	1387 (36.4)	
	8 min~	1073 (26.1)	73 (24.2)	1000 (26.2)	
	Mean (SD)	9.5 (10.7)	10.5 (13.1)	9.3 (10.4)	
Multi-tier response					<0.01
	Yes	2819 (68.5)	232 (76.8)	2587 (67.8)	
Airway management					0.10
	Advanced airway	344 (8.4)	29 (9.6)	315 (8.3)	
	Bag-valve mask	3508 (85.2)	262 (86.8)	3246 (85.1)	
	No airway	263 (6.4)	11 (3.6)	252 (6.6)	
ED level					<0.01
	Level 1 or 2	3397 (82.5)	300 (99.4)	3097 (81.3)	
Hospital treatment					
	ECMO	45 (1.1)	8 (2.6)	37 (1.0)	<0.01
	PCI	323 (7.8)	78 (25.8)	245 (6.4)	<0.01
Outcome					
	Survival to discharge	521 (12.7)	115 (38.1)	406 (10.6)	<0.01
	Good CPC	254 (6.2)	50 (16.6)	204 (5.4)	<0.01

TTM—targeted temperature management, PCI—percutaneous coronary intervention, ECMO—extracorporeal membrane oxygenation, CPC—cerebral performance category.

**Table 2:** Baseline characteristics by percutaneous coronary intervention (PCI).

		Total	PCI		
			Yes	No	*p*-Value
		N (%)		N (%)	
Total		4115 (100.0)	323 (100.0)	3792 (100.0)	
Age					<0.01
	19–65	1174 (28.5)	149 (46.1)	1025 (27.0)	
	65–	2941 (71.5)	174 (53.9)	2767 (73.0)	
Gender					0.49
	Female	1333 (32.4)	99 (30.7)	1234 (32.5)	
Comorbidity					
	Diabetes	1158 (28.1)	119 (36.8)	1039 (27.4)	<0.01
	Hypertension	1548 (37.6)	153 (47.4)	1395 (36.8)	<0.01
	Cardiac comorbidities	745 (18.1)	104 (32.2)	641 (16.9)	<0.01
	Stroke	309 (7.5)	30 (9.3)	279 (7.4)	0.21
	Kidney disease	311 (7.6)	32 (9.9)	279 (7.4)	0.10
Metropolis					0.07
	Yes	1876 (45.6)	163 (50.5)	1713 (45.2)	
Place of arrest					<0.01
	Public	1509 (36.7)	169 (52.3)	1340 (35.3)	
Arrest witnessed					0.02
	Yes	2759 (67.0)	235 (72.8)	2524 (66.6)	
Bystander CPR					<0.01
	Yes	2043 (49.6)	203 (62.8)	1840 (48.5)	
Shockable rhythm at EMS					<0.01
	Shockable	536 (13.0)	186 (57.6)	350 (9.2)	
Response time interval					0.10
	~5 min	926 (22.5)	87 (26.9)	839 (22.1)	
	6~8 min	1627 (39.5)	127 (39.3)	1500 (39.6)	
	9 min~	1562 (38.0)	109 (33.7)	1453 (38.3)	
	Mean (SD)	8.5(4.4)	7.9(3.7)	8.5(4.5)	
Scene time interval					0.05
	~10 min	1097 (26.7)	105 (32.5)	992 (26.2)	
	11~15 min	1429 (34.7)	119 (36.8)	1310 (34.5)	
	16 min~	1589 (38.6)	99 (30.7)	1490 (39.3)	
	Mean (SD)	14.5(6.8)	13.5(5.8)	14.6(6.8)	
Transport time interval					<0.01
	~4 min	1524 (37.0)	105 (32.5)	1419 (37.4)	
	5~7 min	1518 (36.9)	116 (35.9)	1402 (37.0)	
	8 min~	1073 (26.1)	102 (31.6)	971 (25.6)	
	Mean (SD)	9.5 (10.7)	11.3 (12.9)	9.3 (10.4)	
Multi-tier response					0.09
	Yes	2819 (68.5)	235 (72.8)	2584 (68.1)	
Airway management					0.85
	Advanced airway	344 (8.4)	27 (8.4)	317 (8.4)	
	Bag-valve mask	3508 (85.2)	273 (84.5)	3235 (85.3)	
	No airway	263 (6.4)	23 (7.1)	240 (6.3)	
ED level					<0.01
	Level 1 or 2	3397 (82.5)	309 (95.7)	3088 (81.4)	
Hospital treatment					
	ECMO	45 (1.1)	36 (11.1)	9 (0.2)	<0.01
	TTM	302 (7.3)	78 (24.1)	224 (5.9)	<0.01
Outcome					
	Survival to discharge	521 (12.7)	191 (59.1)	330 (8.7)	<0.01
	Good CPC	254 (6.2)	146 (45.2)	108 (2.8)	<0.01

TTM—targeted temperature management, PCI—percutaneous coronary intervention, ECMO—extracorporeal membrane oxygenation, CPC—cerebral performance category.

**Table 3:** Outcomes of post-resuscitation treatments.

Study Outcomes	Total	Outcome		Model 1	Model 2	Model 3
	N	n	%	AOR (95% CI)	AOR (95% CI)	AOR (95% CI)
Survival to discharge						
TTM (−)	3813	406	10.65	reference	reference	reference
TTM (+)	302	115	38.08	4.83 (3.73–6.27) *	4.47 (3.44–5.81) *	3.91 (2.92–5.22) *
PCI (−)	3792	330	8.70	reference	reference	reference
PCI (+)	323	191	59.13	14.18 (11.02–18.25) *	13.38 (10.34–17.31) *	7.95 (5.97–10.57) *
Good neurological recovery						
TTM (−)	3813	204	5.35	reference	reference	reference
TTM (+)	302	50	16.56	2.94 (2.08–4.16) *	2.68 (1.88–3.82) *	1.69 (1.12–2.55) *
PCI (−)	3792	108	2.85	reference	reference	reference
PCI (+)	323	146	45.20	25.85 (19.11–34.98) *	24.47 (17.91–33.43) *	11.35 (7.98–16.14) *

* Statistically significant at *p* < 0.05; Model 1: adjusted for age, sex, metropolis; Model 2: adjusted for variables in model 1 and diabetes mellitus, hypertension, cardiac comorbidities, stroke, kidney disease; Model 3: adjusted for variables in model 2 and bystander CPR, witnessed, place of cardiac arrest private place, shockable rhythm at EMS; AOR— adjusted odds ratio CI—confidence interval, TTM—targeted temperature management, PCI—percutaneous coronary intervention.

**Table 4:** Logistic regression analysis for comorbidities.

Study Outcomes	Total	Outcome		Model 1	Model 2	Model 3
	N	n	%	AOR (95% CI)	AOR (95% CI)	AOR (95% CI)
Survival to discharge						
DM (−)	2957	377	12.75	reference	reference	reference
DM (+)	1158	144	12.44	1.10 (0.89–1.35)	0.84 (0.67–1.06)	0.93 (0.73–1.19)
HTN (−)	2567	295	11.49	reference	reference	reference
HTN (+)	1548	226	14.60	1.59 (1.31–1.93) *	1.56 (1.26–1.93) *	1.46 (1.16–1.83) *
Good neurological recovery						
DM (−)	2957	188	6.36	reference	reference	reference
DM (+)	1158	66	5.70	1.13 (0.84–1.52)	0.85 (0.61–1.18)	1.00 (0.69–1.43)
HTN (−)	2567	151	5.88	reference	reference	reference
HTN (+)	1548	103	6.65	1.63 (1.24–2.14) *	1.62 (1.20–2.18) *	1.48 (1.06–2.06) *

* Statistically significant at *p* < 0.05; Model 1: adjusted for age, sex, metropolis; Model 2: adjusted for variables in model 1 and diabetes mellitus, hypertension, cardiac comorbidities, stroke, kidney disease; Model 3: adjusted for variables in model 2 and bystander CPR, witnessed, place of cardiac arrest private place, shockable rhythm at EMS; AOR—adjusted odds ratio CI—confidence interval, HTN—hypertension, DM—diabetes mellitus.

**Table 5:** Logistic regression analysis of interactions between post-resuscitation treatments and comorbidities, with FDR-adjusted *p*-values.

**Survival to Discharge**	**TTM (−)**	**TTM (+)**	**Adjusted *p*-for Interaction**	**PCI (−)**	**PCI (+)**	**Adjusted *p*-for Interaction**
		**AOR (95% CI)**			**AOR (95% CI)**	
DM (−)	reference	4.91 (3.49–6.91)	<0.05	reference	10.33 (7.23–14.76)	<0.05
DM (+)	reference	2.20 (1.26–3.86)		reference	5.00 (3.14–7.96)	
HTN (−)	reference	6.05 (4.10–8.91)	<0.05	reference	9.97 (6.77–14.68)	0.16
HTN (+)	reference	2.31 (1.48–3.58)		reference	6.18 (4.13–9.25)	
**Good Neurological Recovery**	**TTM (−)**	**TTM (+)**	**Adjusted *p*-for Interaction**	**PCI (−)**	**PCI (+)**	**Adjusted *p*-for Interaction**
		**AOR (95% CI)**			**AOR** **(95% CI)**	
DM (−)	reference	2.32 (1.46–3.69)	<0.05	reference	16.45 (10.80–25.05)	<0.05
DM (+)	reference	0.59 (0.23–1.49)		reference	4.94 (2.69–9.06)	
HTN (−)	reference	2.58 (1.52–4.38)	<0.05	reference	15.77 (9.94–25.02)	<0.05
HTN (+)	reference	0.94 (0.49–1.82)		reference	7.47 (4.48–12.44)	

Adjusted for age, sex, metropolis, diabetes mellitus, hypertension, cardiac comorbidities, stroke, kidney disease, bystander CPR, witnessed, place of cardiac arrest private place, shockable rhythm at EMS; adjusted *p*-values for interaction terms were calculated using the Benjamini–Hochberg method to control the false discovery rate; AOR—adjusted odds ratio CI—confidence interval, HTN—hypertension, DM—diabetes mellitus, TTM—targeted temperature management, PCI—percutaneous coronary intervention.

## Data Availability

The datasets collected and analyzed in the current study are available from the corresponding author on reasonable request.

## Abbreviations

The following abbreviations are used in this manuscript:

OHCA	Out-of-hospital cardiac arrest
EMS	Emergency medical service
TTM	Targeted temperature management
PCI	Percutaneous coronary intervention
DM	Diabetes mellitus
HTN	Hypertension
ROSC	Return of spontaneous circulation
CPC	Cerebral performance category
ECMO	Extracorporeal membrane oxygenation
ED	Emergency department
CAG	Coronary angiography
CVD	Cardiovascular disease
CI	Confidence interval
aOR	Adjusted odds ratio
CPR	Cardiopulmonary resuscitation
CDC	Centers for Disease Control and Prevention
IRB	Institutional Review Board

## References

[1] Kiyohara K., Katayama Y., Kitamura T., Kiguchi T., Matsuyama T., Ishida K., Sado J., Hirose T., Hayashida S., Nishiyama C. (2020). Gender disparities in the application of public-access AED pads among OHCA patients in public locations. Resuscitation.

[2] Tsao C.W., Aday A.W., Almarzooq Z.I., Anderson C.A.M., Arora P., Avery C.L., Baker-Smith C.M., Beaton A.Z., Boehme A.K., Buxton A.E. (2023). Heart Disease and Stroke Statistics-2023 Update: A Report From the American Heart Association. Circulation.

[3] Nishiyama C., Kiguchi T., Okubo M., Alihodžić H., Al-Araji R., Baldi E., Beganton F., Booth S., Bray J., Christensen E. (2023). Three-year trends in out-of-hospital cardiac arrest across the world: Second report from the International Liaison Committee on Resuscitation (ILCOR). Resuscitation.

[4] Bougouin W., Mustafic H., Marijon E., Murad M.H., Dumas F., Barbouttis A., Jabre P., Beganton F., Empana J.P., Celermajer D.S. (2015). Gender and survival after sudden cardiac arrest: A systematic review and meta-analysis. Resuscitation.

[5] Sasson C., Rogers M.A., Dahl J., Kellermann A.L. (2010). Predictors of survival from out-of-hospital cardiac arrest: A systematic review and meta-analysis. Circ. Cardiovasc. Qual. Outcomes.

[6] Sanna T., La Torre G., de Waure C., Scapigliati A., Ricciardi W., Dello Russo A., Pelargonio G., Casella M., Bellocci F. (2008). Cardiopulmonary resuscitation alone vs. cardiopulmonary resuscitation plus automated external defibrillator use by non-healthcare professionals: A meta-analysis on 1583 cases of out-of-hospital cardiac arrest. Resuscitation.

[7] Bernard S.A., Gray T.W., Buist M.D., Jones B.M., Silvester W., Gutteridge G., Smith K. (2002). Treatment of comatose survivors of out-of-hospital cardiac arrest with induced hypothermia. N. Engl. J. Med..

[8] Morrison L.J., Neumar R.W., Zimmerman J.L., Link M.S., Newby L.K., McMullan P.W., Hoek T.V., Halverson C.C., Doering L., Peberdy M.A. (2013). Strategies for improving survival after in-hospital cardiac arrest in the United States: 2013 consensus recommendations: A consensus statement from the American Heart Association. Circulation.

[9] Vyas A., Chan P.S., Cram P., Nallamothu B.K., McNally B., Girotra S. (2015). Early Coronary Angiography and Survival After Out-of-Hospital Cardiac Arrest. Circ. Cardiovasc. Interv..

[10] (2017). Global, regional, and national under-5 mortality, adult mortality, age-specific mortality, and life expectancy, 1970-–2016: A systematic analysis for the Global Burden of Disease Study 2016. Lancet.

[11] Jemal A., Ward E.M., Johnson C.J., Cronin K.A., Ma J., Ryerson B., Mariotto A., Lake A.J., Wilson R., Sherman R.L. (2017). Annual Report to the Nation on the Status of Cancer, 1975-2014, Featuring Survival. J. Natl. Cancer Inst..

[12] Winther-Jensen M., Kjaergaard J., Hassager C., Køber L., Lippert F., Søholm H. (2020). Cancer is not associated with higher short or long-term mortality after successful resuscitation from out-of-hospital cardiac arrest when adjusting for prognostic factors. Eur. Heart J. Acute Cardiovasc. Care.

[13] Kang S.B., Kong S.Y.J., Shin S.D., Ro Y.S., Song K.J., Hong K.J., Kim T.H. (2019). Effect of cancer history on post-resuscitation treatments in out-of-hospital cardiac arrest. Resuscitation.

[14] Kim J.W., Monlezun D., Park J.K., Chauhan S., Balanescu D., Koutroumpakis E., Palaskas N., Kim P., Hassan S., Botz G. (2022). Post-cardiac arrest PCI is underutilized among cancer patients: Machine learning augmented nationally representative case-control study of 30 million hospitalizations. Resuscitation.

[15] Guha A., Buck B., Biersmith M., Arora S., Yildiz V., Wei L., Awan F., Woyach J., Lopez-Mattei J., Plana-Gomez J.C. (2019). Contemporary impacts of a cancer diagnosis on survival following in-hospital cardiac arrest. Resuscitation.

[16] Fowler H., Belot A., Ellis L., Maringe C., Luque-Fernandez M.A., Njagi E.N., Navani N., Sarfati D., Rachet B. (2020). Comorbidity prevalence among cancer patients: A population-based cohort study of four cancers. BMC Cancer.

[17] Kim S.S., Kim H.S. (2022). The Impact of the Association between Cancer and Diabetes Mellitus on Mortality. J. Pers. Med..

[18] Kim Y.G., Roh S.Y., Han K.D., Jeong J.H., Choi Y.Y., Min K., Shim J., Choi J.I., Kim Y.H. (2022). Hypertension and diabetes including their earlier stage are associated with increased risk of sudden cardiac arrest. Sci. Rep..

[19] Oh S., Lee J., Hong Y.S., Kim K. (2023). Increased risk of cardiovascular disease associated with diabetes among adult cancer survivors: A population-based matched cohort study. Eur. J. Prev. Cardiol..

[20] Jung E., Park J.H., Ro Y.S., Song K.J., Ryu H.H., Lee S.C., Shin S.D. (2019). Effect of hypertension across the age group on survival outcomes in out-of-hospital cardiac arrest. Am. J. Emerg. Med..

[21] Ro Y.S., Shin S.D., Song K.J., Lee E.J., Lee Y.J., Kim J.Y., Jang D.B., Kim M.J., Kong S.Y. (2015). Interaction effects between hypothermia and diabetes mellitus on survival outcomes after out-of-hospital cardiac arrest. Resuscitation.

[22] Daviaud F., Dumas F., Demars N., Geri G., Bouglé A., Morichau-Beauchant T., Nguyen Y.L., Bougouin W., Pène F., Charpentier J. (2014). Blood glucose level and outcome after cardiac arrest: Insights from a large registry in the hypothermia era. Intensive Care Med..

[23] Lee B.K., Lee H.Y., Jeung K.W., Jung Y.H., Lee G.S., You Y. (2013). Association of blood glucose variability with outcomes in comatose cardiac arrest survivors treated with therapeutic hypothermia. Am. J. Emerg. Med..

[24] Kim K.H., Ro Y.S., Park J.H., Kim T.H., Jeong J., Hong K.J., Song K.J., Shin S.D. (2021). Association between case volume of ambulance stations and clinical outcomes of out-of-hospital cardiac arrest: A nationwide multilevel analysis. Resuscitation.

[25] The Brain Resuscitation Clinical Trial II Study Group (1991). A randomized clinical trial of calcium entry blocker administration to comatose survivors of cardiac arrest. Design, methods, and patient characteristics. Control. Clin. Trials.

[26] Benjamini Y., Hochberg Y. (2018). Controlling the False Discovery Rate: A Practical and Powerful Approach to Multiple Testing. J. R. Stat. Soc. Ser. B Methodol..

[27] Domanski M.J., Exner D.V., Borkowf C.B., Geller N.L., Rosenberg Y., Pfeffer M.A. (1999). Effect of angiotensin converting enzyme inhibition on sudden cardiac death in patients following acute myocardial infarction. A meta-analysis of randomized clinical trials. J. Am. Coll. Cardiol..

[28] Jefferies J.L., Mazur W.M., Howell C.R., Plana J.C., Ness K.K., Li Z., Joshi V.M., Green D.M., Mulrooney D.A., Towbin J.A. (2021). Cardiac remodeling after anthracycline and radiotherapy exposure in adult survivors of childhood cancer: A report from the St Jude Lifetime Cohort Study. Cancer.

[29] Dong H., Yao L., Wang M., Wang M., Li X., Sun X., Yu X., Guo J., Li X., Xu Y. (2020). Can ACEI/ARB prevent the cardiotoxicity caused by chemotherapy in early-stage breast cancer?-a meta-analysis of randomized controlled trials. Transl. Cancer Res..

[30] Simek S., Motovska Z., Hlinomaz O., Kala P., Hromadka M., Knot J., Varvarovsky I., Dusek J., Rokyta R., Tousek F. (2020). The Effect of Diabetes on Prognosis Following Myocardial Infarction Treated with Primary Angioplasty and Potent Antiplatelet Therapy. J. Clin. Med..

[31] Lin M.J., Chen C.Y., Lin H.D., Wu H.P. (2017). Impact of diabetes and hypertension on cardiovascular outcomes in patients with coronary artery disease receiving percutaneous coronary intervention. BMC Cardiovasc. Disord..

[32] Kini A.S., Kim M.C., Moreno P.R., Krishnan P., Ivan O.C., Sharma S.K. (2008). Comparison of coronary flow reserve and fractional flow reserve in patients with versus without diabetes mellitus and having elective percutaneous coronary intervention and abciximab therapy (from the PREDICT Trial). Am. J. Cardiol..

[33] Jung E., Lee S.Y., Park J.H., Ro Y.S., Hong K.J., Song K.J., Ryu H.H., Shin S.D. (2020). Interaction Effects Between Targeted Temperature Management and Hypertension on Survival Outcomes After Out-of-Hospital Cardiac Arrest: A National Observational Study from 2009 to 2016. Ther. Hypothermia Temp. Manag..

[34] Pires P.W., Dams Ramos C.M., Matin N., Dorrance A.M. (2013). The effects of hypertension on the cerebral circulation. Am. J. Physiol. Heart Circ. Physiol..

[35] Donnino M.W., Andersen L.W., Berg K.M., Reynolds J.C., Nolan J.P., Morley P.T., Lang E., Cocchi M.N., Xanthos T., Callaway C.W. (2015). Temperature Management After Cardiac Arrest: An Advisory Statement by the Advanced Life Support Task Force of the International Liaison Committee on Resuscitation and the American Heart Association Emergency Cardiovascular Care Committee and the Council on Cardiopulmonary, Critical Care, Perioperative and Resuscitation. Circulation.

[36] Shenasa M., Shenasa H. (2017). Hypertension, left ventricular hypertrophy, and sudden cardiac death. Int. J. Cardiol..

[37] Messerli F.H., Rimoldi S.F., Bangalore S. (2017). The Transition From Hypertension to Heart Failure: Contemporary Update. JACC Heart Fail..

[38] Sarfati D., Koczwara B., Jackson C. (2016). The impact of comorbidity on cancer and its treatment. CA Cancer J. Clin..

[39] Arreskov A.B., Olsen M., Pouplier S.S., Siersma V., Andersen C.L., Friis S., de Fine Olivarius N. (2019). The impact of cancer on diabetes outcomes. BMC Endocr. Disord..

[40] Roxburgh C.S., McMillan D.C. (2014). Cancer and systemic inflammation: Treat the tumour and treat the host. Br. J. Cancer.

[41] Elezaby A., Dexheimer R., Sallam K. (2022). Cardiovascular effects of immunosuppression agents. Front. Cardiovasc. Med..

[42] Boicean A., Ichim C., Sasu S.M., Todor S.B. (2025). Key Insights into Gut Alterations in Metabolic Syndrome. J. Clin. Med..

[43] Ichim C., Pavel V., Mester P., Schmid S., Todor S.B., Stoia O., Anderco P., Kandulski A., Müller M., Heumann P. (2024). Assessing Key Factors Influencing Successful Resuscitation Outcomes in Out-of-Hospital Cardiac Arrest (OHCA). J. Clin. Med..

